# RNA-Seq Analysis of the Response of the Halophyte, *Mesembryanthemum crystallinum* (Ice Plant) to High Salinity

**DOI:** 10.1371/journal.pone.0118339

**Published:** 2015-02-23

**Authors:** Hironaka Tsukagoshi, Takamasa Suzuki, Kouki Nishikawa, Sakae Agarie, Sumie Ishiguro, Tetsuya Higashiyama

**Affiliations:** 1 The Center for Gene Research, Nagoya University, Furo-cho, Chikusa-ku, Nagoya, Aichi, Japan; 2 Program for leading graduate schools, PhD Professional: Gateway to Success in Frontier Asia, Nagoya University, Furo-cho, Chikusa-ku, Nagoya, Aichi, Japan; 3 PRESTO, JST, Honcho, Kawaguchi, Saitama, Japan; 4 Division of Biological Science, Graduate School of Science, Nagoya University, Furo-cho, Chikusa-ku, Nagoya, Aichi, Japan; 5 JST, ERATO, Higashiyama Live-Holonics Project, Nagoya University, Furo-cho, Chikusa-ku, Nagoya, Aichi, Japan; 6 Graduate School of Bioagricultural Sciences, Nagoya University, Furo-cho, Chikusa-ku, Nagoya, Aichi, Japan; 7 Faculty of Agriculture, Kagawa university, Saiwaicho 1-1, Takamatsu, Kagawa, Japan; 8 WPI-ITbM, Nagoya University, Furo-cho, Chikusa-ku, Nagoya, Aichi, Japan; University of Minho, PORTUGAL

## Abstract

Understanding the molecular mechanisms that convey salt tolerance in plants is a crucial issue for increasing crop yield. The ice plant (*Mesembryanthemum crystallinum*) is a halophyte that is capable of growing under high salt conditions. For example, the roots of ice plant seedlings continue to grow in 140 mM NaCl, a salt concentration that completely inhibits *Arabidopsis thaliana* root growth. Identifying the molecular mechanisms responsible for this high level of salt tolerance in a halophyte has the potential of revealing tolerance mechanisms that have been evolutionarily successful. In the present study, deep sequencing (RNAseq) was used to examine gene expression in ice plant roots treated with various concentrations of NaCl. Sequencing resulted in the identification of 53,516 contigs, 10,818 of which were orthologs of *Arabidopsis* genes. In addition to the expression analysis, a web-based ice plant database was constructed that allows broad public access to the data. The results obtained from an analysis of the RNAseq data were confirmed by RT-qPCR. Novel patterns of gene expression in response to high salinity within 24 hours were identified in the ice plant when the RNAseq data from the ice plant was compared to gene expression data obtained from *Arabidopsis* plants exposed to high salt. Although ABA responsive genes and a sodium transporter protein (HKT1), are up-regulated and down-regulated respectively in both *Arabidopsis* and the ice plant; peroxidase genes exhibit opposite responses. The results of this study provide an important first step towards analyzing environmental tolerance mechanisms in a non-model organism and provide a useful dataset for predicting novel gene functions.

## Introduction

High salinity is a critical problem in crop production that results in reduced plant growth and a significant reduction in productivity. The amount of arable land impacted by high salinity has increased, due to climate change, irrigation practices, desertification, flood, and other causes. The Food and Agriculture Organization of the United Nations (FAO) estimated that 45 million ha out of 230 million ha of irrigated land is affected by salinity (FAO: http://www.fao.org/home/en/). Studies using *Arabidopsis* as a model plant have identified a number of genes involved in salt tolerance. In particular, several transcription factors have been identified as key regulators of salt tolerance in *Arabidopsis*, such as DREB2A [1]. Additionally, DREB2A orthologs in other plant species, such as rice, soybean, poplar, buffalograss, and sugarcane, also appear to be involved in salt tolerance [2], [3], [4], [5], [6]. Collectively, these studies have demonstrated that the DREB2A gene regulatory network is an important molecular mechanism for salt tolerance in the Plant Kingdom. Additional data from *Arabidopsis* have also revealed cross-talk of the DREB2A pathway with other pathways, such as the ABA-mediated signaling, osmotic response, and some ionic response pathways that are induced by exposure to high salt [1].

It is commonly accepted that better root growth supports better whole plant growth. Since root growth is strongly inhibited under high salt conditions, understanding how roots respond to high levels of salt is essential to understand salt tolerance. Numerous studies have been conducted at the molecular level on the response of *Arabidopsis* roots to high salt conditions. Dinneny et al. [7] reported that cell-type specific salt response machinery is essential for determining the appropriate transcriptional response to salt stress. Morphological changes in the root, such as swollen cortical cells and a delay in root hair development are among the cell-type specific responses to high salt. These changes have been shown by using live-imaging analysis and these responses occurred within 24 hours in the roots of *Arabidopsis thaliana* [8]. In addition to the information derived from the studies of cell type–specificity, the analysis of salt tolerance among naturally occurring genetic variants (accessions) of *Arabidopsis* has also provided important molecular information. Katori et al. [9], in a study of *Arabidopsis* accessions, identified several QTLs that were associated with salt tolerance. Importantly, a genome wide association study (GWAS) indicated that the ability to accumulate NaCl in the leaves of *Arabidopsis* is dependent on the genetic variation of the Na transporter, AtHKT1 [10]. The authors indicated that the genetic variation is most likely related to the adaptation to coastal or high saline soil environments [10].

With the recent advances in sequencing and bioinformatic technologies, researchers have begun to move to non-model plants to study molecular mechanisms that are responsible for salt tolerance. *Thellungiella halophila* has been widely used due the similarity of its genome sequence to *Arabidopsis*. A recent study has also reported on the transcriptional response to high levels of salinity in semi-mangrove plants [11]. Using deep sequencing technology, Huang et al. [11] reported on gene expression in response to salt that was partially common to a variety of plants and species-specific responses also existed. Their study demonstrates the ability to use non-model plants to address biological questions. Based on this premise, we propose that studying gene expression in halophytic plants can discover unique aspects of salt tolerance.

*Mesembryanthemum crystallinum* (ice plant) is a halophyte that switches from C3 photosynthesis to Crassulacean acid metabolism (CAM) under high salinity and drought stress (reviewed in [12]). Mature ice plants can grow in soil that contains a salt concentration above 450 mM NaCl, which is higher than found within seawater [11]. This finding was based on studying the response of shoot growth to high salinity, however, to date no studies have been conducted in ice plants to characterize the response of roots to high salt concentrations. The genome size of *M. crystallinum* is 250 to 300 M bp and comprised of 2n = 18 chromosomes [13], [14]. Although transformation technologies for ice plant have not been established, ice plant seedlings are similar in size to *Arabidopsis* making their use in molecular analysis relatively straightforward. Since roots directly contact the soil containing the high concentrations of salt, analyzing root growth in this halophyte and their molecular response to high salinity should provide significant insight into the molecular adaptation of roots to high levels of salt. Genes identified in *M. crystallinum* that are associated with salt tolerance will serve as strong candidates for use in the genetic engineering of agricultural crops with increased salt tolerance.

In the present study, deep sequencing technology was used to characterize the regulatory network underlying high salinity tolerance in the ice plant. The transcriptomic dataset obtained from *M. crystallinum* was used to construct an ice plant mRNA database. Using this database, the transcriptional responses of ice plant and *Arabidopsis* were compared in order to determine if essential salt response pathways are conserved in these plant species. These data sets can be used to investigate the molecular mechanism of short-term salt tolerance in a non-model plant and should provide new insight into salt tolerance. The information obtained from this study, and the identified genes associated with salt tolerance, can be used to advance efforts to use plant biotechnology to improve agricultural productivity.

## Materials and Methods

### Plant growth and salt treatment

Seeds of *M. crystallinum* and *Arabidopsis thaliana* Col-0 ecotype were maintained in the dark at 4°C for 1 day, sterilized for 5 min in 25% bleach and 0.05% Triton X-100, washed 3 times with sterile water and sown onto Murashige-Skoog (MS) medium (pH 5.8) containing 1% sucrose and 1% agarose. Seeds were germinated in a vertical orientation for 5 days in a growth chamber at 22°C with a 16 h light and 8 h dark light regime (light intensity of 65 μmol Photons m^-2^sec^-1^).

For NaCl treatment, five-day-old seedlings were transferred onto MS media containing either 140 mM, 250 mM, or 500 mM NaCl for 24 h. The plants were imaged under a stereomicroscope (Olympus SZX12) with a DP70 CCD camera. Root length was measured using Image-J software (http://imagej.net/).

### RNA extraction and deep sequencing

Whole roots from five-day-old seedlings treated with 0 mM, 140 mM, 250 mM, or 500 mM NaCl for 24 h were used for RNA extraction with an RNeasy plant mini kit (Qiagen) according to the manufacturer’s instructions. A single RNA isolate that was pooled from 20 roots was used for deep sequencing analysis and three biological replicates were utilized for RT-qPCR experiments.

A TruSeq RNA Sample Preparation kit (Illumina) was used to construct cDNA libraries according to the manufacturer’s instructions. Briefly, 2 μg of total RNA were used for polyA selections with RNA purification beads. The cDNA library was purified by AMPure (Beckman coulter) by using a magnetic stand. The length of the cDNAs was determined with an Agilent Technologies 2100 Bioanalyzer using the Agilent DNA 1000 chip kit and cDNA quantity was measured by qPCR using PhiX Control (Illumina) as a standard. Both the 5’ and 3’ ends of the cDNAs were sequenced using an Illumina Genome Analyzer IIx with a paired end module for 60 cycles (Illumina). The resulting sequence data were deposited in the DDBJ Sequence Read Archive (DRA) at the DNA Data Bank of Japan (DDBJ; http://www.ddbj.nig.ac.jp/) under the accession number, DRP002316.

### *De novo* assembly and annotation

A total of 84 million paired-reads from four libraries were filtered using cutadapt [15]. Low quality reads, which contained more than 20 nucleotides with less than a 15 quality value were further filtered. The remaining 70 million reads were used in the *de novo* assembly with Trinity [16] software released at 2013_08_14 with the following options “—seqType fq—output working_dir—CPU 4—JM 100G—left left.fastq—right right.fastq”. A total of 53,516 contigs were obtained (The assembled sequences can also be found in the DDBJ data libraries with accession numbers FX891461-FX944976). Using blastx, all contigs were queried against the *A. thaliana* protein database (TAIR10, http://www.arabidopsis.org/) in order to annotate them and identify the open reading frame. A total of 31,733 contigs, out of 53,516 contigs, had homology to genes in *Arabidopsis* and were grouped into 13,855 genes in *Arabidopsis*. A reciprocal blast search, namely tblastn search using Arabidopsis proteome as queries against ice plant contigs, were perfomed we cut off the result more than 1e^-3^ of e-value. A total of 10,818 pairs were selected as orthologous genes.

### Data analysis

The first read of the paired end reads were used to analyze gene expression. Low quality reads, which contained more than 20 nucleotides with less than 15 quality value, were discarded prior to mapping. The filtered reads were mapped to the assembled 53,516 contigs using Bowtie [17] software and the number of reads mapping to each contig was counted. By using 10,818 orthologous genes, we identified differentially expressed genes with the R package, DESeq [18]. We used the following cut off values to determine differentially expressed genes between the control (0 mM NaCl) and treated samples: FDR<0.05 and |Fold Change (FC)|>2.

We performed a Gene Ontology (GO) analysis for biological functions by using these differentially expressed genes. GO enrichment categories of expression analysis were identified by using ChipEnrich software [19], which was available from http://www.arexdb.org/software.jsp. GO enrichment analysis associates each gene of a list with different biological processes and then subsequently evaluates whether the list contains more genes than expected “by chance” for a certain biological process.

### Database construction

The database was built on a home-made cluster computer named Kiku 1st. Linux (http://www.centos.org/) and PostgreSQL (http://www.postgresql.org/) were installed as an operating system and as a relational database management system, respectively. The web interface was developed with PHP (http://www.php.net/) and ZendFramework libraries (http://framework.zend.com/), which were run on an Apache (http://httpd.apache.org/) web server.

### RT-qPCR

First strand cDNA was synthesized by using the PrimeScript RT reagent Kit with gDNA Eraser (TAKARA). Reverse transcription—quantitative PCR (RT-qPCR) was performed using THUNDERBIRD SYBR qPCR Mix (TOYOBO) on an ABI 7500 Real-Time PCR (Applied Biosystems). RT-qPCR reactions were performed in a total volume of 25 μl; with 1 μl of first-strand cDNAs and 1 μl of each primer. The cycler conditions were: 1 min at 95°C, followed by 40 cycle of 15 sec at 95°C and 35 sec at 60°C. The primers that were used in this study are listed in S4 Table.

RT-qPCR efficiency and the CT values for individual reactions were determined by the analysis of raw fluorescent data using the free web based algorithm PCR Miner [20] (http://www.miner.ewindup.info). Efficiency corrected transcript abundance values of three biological replicates were used for determining the relative expression values for all samples. Normalization of mRNA levels was performed against the level of poly *UBQ10* mRNA as previously described [21]. Statistical significance was evaluated using a Student’s *t* test analysis with and excel plugin “StatPlus”. Primer specificity was confirmed by measuring the melting curve analysis after 40 amplification cycles by increasing the temperature from 60°C to 95°C.

## Results

### Ice plant roots are tolerant to salt concentrations that inhibit *Arabidopsis* root growth

**Fig 1:**
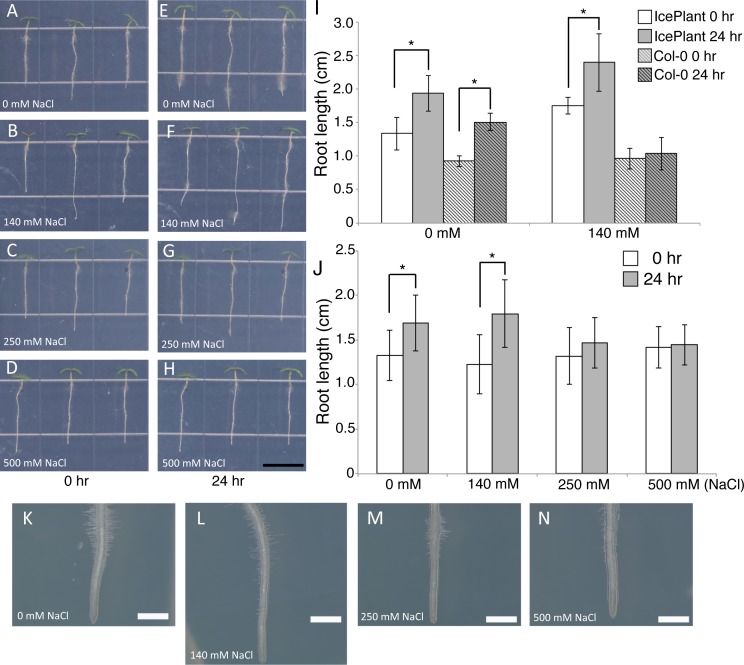
Effect of NaCl on root growth in ice plants. (A-D) Five-day-old ice plant seedlings grown on the MS medium prior to NaCl treatment. (E-H) five-day-old ice plant seedlings after 24 h on MS containing 0 mM NaCl (E), 140 mM NaCl (F), 250 mM NaCl (G), or 500 mM NaCl (H). Bar = 1 cm. (I) Root length in five-day-old ice plant and *Arabidopsis thaliana* Col-0 ecotype seedlings (white boxes and white boxes with diagonal lines) and that of the same roots after 24 h on plates containing 140 mM NaCl (gray boxes and gray boxes with diagonal lines). Mean ± standard deviation (SD) (n = 15): *p<0.001, as determined by a Student’s *t*-test. (J) Root length in five-day-old ice plants (white boxes) and that of the same roots after 24 h on the indicated concentration of NaCl (gray boxes). Mean ± standard deviation (SD) (n = 15): *p<0.001, as determined by a Student’s *t*-test. (K-N) Morphological changes in root hairs on roots of ice plant seedlings treated with 0 mM (K), 140 mM (L), 250 mM (M) or 500 mM NaCl (N) for 24 h. Bar = 1 mm.

Five-day-old seedlings of ice plant and *Arabidopsis*, Col-0 accession, were treated with 0 and 140 mM NaCl for 24 h, after which time root length was measured (Fig. 1A-H). Root growth of the *Arabidopsis* Col-0 accession was completely inhibited by 140 mM NaCl treatment for 24 h. In contrast, 140 mM NaCl treatment did not inhibit ice plant root growth (Fig. 1I). This result indicated that ice plant has a greater tolerance to NaCl stress than *Arabidopsis*. The concentration of NaCl was then increased to determine whether or not ice plant is tolerant to a higher concentration of NaCl. Results indicated that 250 and 500 mM NaCl both strongly inhibited root growth in *M. crystallinum* (Fig. 1J). As a point of reference, 500 mM NaCl is higher than the concentration of NaCl that is typically found in seawater (450 mM, [11]). In addition to the inhibition of primary root growth, the high salt concentrations also inhibited root hair growth in *M. crystallinum* (Fig. 1K to N). Inhibition of root hair growth has also been observed in *Arabidopsis* roots under high salt stress [7]. These results indicate that, although ice plant is tolerant to higher salt concentrations than *Arabidopsis*, similar morphological changes in roots are observed in both species when they are subjected to high salt conditions.

### RNAseq analysis and *de novo* assembly

**Fig 2:**
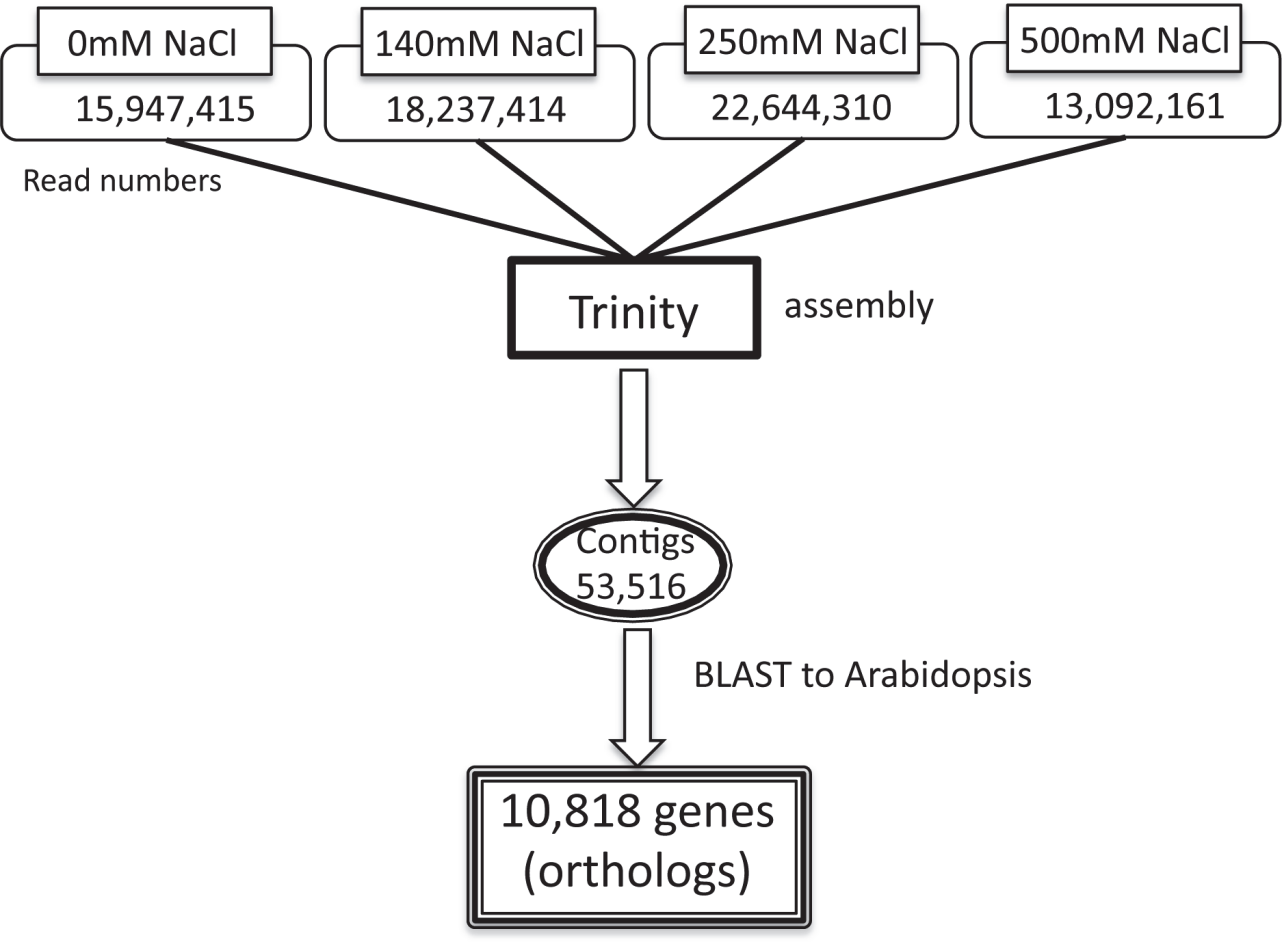
Flowchart of the experimental design used to obtain transcriptome data of the response of ice plant to salt stress. Trinity software was used for the assembly and blastx against the *Arabidopsis* protein database was used for annotation of the assembled ice plant genes.

We were very interested to characterize the transcriptional events involved in the development of salt tolerance in *M. crystallinum*. Therefore, the transcriptional changes in young ice plant roots subjected to various salt concentrations were investigated using high-throughput sequencing technology. Total RNA was isolated from whole roots of five-day-old ice plant seedlings treated with 0 mM, 140 mM, 250 mM, or 500 mM of NaCl for 24 h. The isolated total RNAs were converted to cDNA libraries, and both ends of the cDNAs were sequenced for 60 cycles using a paired-end module. Approximately 84 million paired-reads, 5 G bp in total, were sequenced from four libraries and all reads were assembled using the Trinity software [16]. This resulted in 53,516 contigs, containing 67 M bp sequences. The averages, median and maximum N50 and N90 lengths of the assembled contigs were 1,179 bp, 803 bp and 16,785 bp, 1,919 bp and 518 bp, respectively. To annotate the contigs, the consensus sequence of all the contigs were used as queries against the *Arabidopsis* protein database (TAIR10, http://www.arabidopsis.org/) using blastx. Out of a total of 53,516 contigs, 31,733 contigs had 13,855 homologous genes in *Arabidopsis*. A reciprocal blast search was also performed using Arabidopsis proteome as queries against ice plant contigs and we obtained mutually top hit 10,818 pairs as orthologous genes. These orthologous genes were used as a reference for further analysis (Fig. 2).

The first end read of the paired end reads was mapped to the reference set of contigs using Bowtie [17] software with about 94% of the reads being mapped. The number of mapped reads obtained from the cDNA libraries from roots treated with 0 mM, 140 mM, 250 mM, and 500 mM salt were 8.6, 9.9, 12.2, and 7.0 million reads, respectively. The number of reads mapping to each contig was counted and used to obtain the gene expression data. The results, including the paired end reads, the assembled sequences, and the expression data (S1 Table), are available on the database website (http://dandelion.liveholonics.com/pothos/Mcr/).

### Annotation of genes and comparisons of ice plant gene expression data to gene expression data from microarray datasets of *Arabidopsis* treated with NaCl

**Fig 3:**
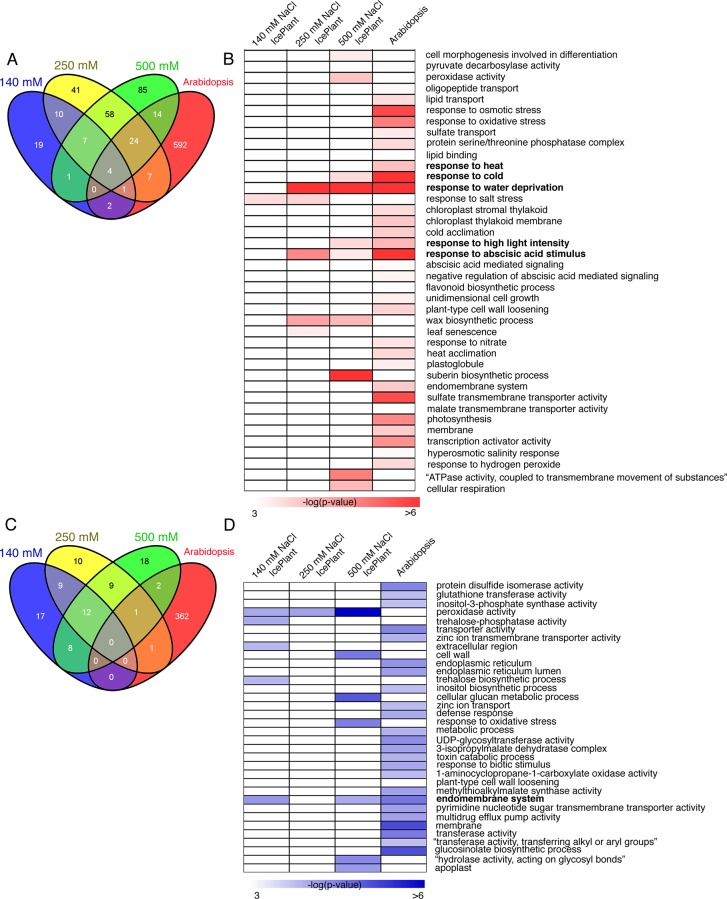
Comparison of salt-responsive genes in *Arabidopsis* and ice plant. Venn diagram of the genes that were up-regulated in *Arabidopsis* (obtained from microarray data) and ice plant (obtained from RNAseq data) (A). Enriched Gene Ontology (GO) categories of biological functions within the list of genes found to be significantly up-regulated by NaCl treatment (B). Venn diagram of the genes that were down-regulated in *Arabidopsis* and ice plant (C). GO categories of biological functions within the list of genes found to be significantly down-regulated by NaCl treatment (D).

To discover which genes in the ice plant were responsive to salt stress, a comparison of the data set obtained from the ice plant with *Arabidopsis* datasets was performed. The ice plant data sets were normalized and a False Discovery Rate (FDR) and fold changes (FC) were calculated using the DESeq package for R [18]. The NaCl-treated datasets were compared to the 0 mM NaCl-treated dataset, the latter of which was considered as a control. A cut off of FDR<0.05 and |FC|>2 was used. Using these criteria, 44, 152, and 193 genes were found to be significantly up-regulated in roots of ice plant in response to 140 mM, 250 mM, and 500 mM NaCl, respectively. The microarray datasets retrieved for *Arabidopsis* from the Gene Expression Omnibus (GEO) database (http://www.ncbi.nlm.nih.gov/gds/; GSM184925.CEL, GSM184926.CEL, GSM184933.CEL, and GSM184934.CEL) for comparison with the ice plant datasets were of five-day-old *Arabidopsis* roots treated with 140 mM NaCl for 16h. The *Arabidopsis* microarray datasets were normalized using gcRMA [22], and FDR and FC were calculated using the SAMr algorithm (S2 Table, [23]). Using the same criteria that were employed on the datasets obtained from the ice plant, 644 genes were found to be significantly up-regulated in *Arabidopsis* roots. Among the up-regulated genes, only 4 genes were common to all datasets (Fig. 3A). On the other hand, 46, 42, 50, and 366 genes were significantly down-regulated in 140 mM, 250 mM, and 500 mM NaCl-treated roots of ice plant, and 140 mM NaCl-treated roots of *Arabidopsis* respectively. Interestingly, no genes were found to be present in all the down-regulated datasets (Fig. 3C).

We subsequently determined whether or not common gene ontology (GO) categories of biological functions could be identified among salt responsive genes in *M. crystallinum* and *Arabidopsis*. A GO analysis was performed using a chip-enrichment program [19]. Only two enriched GO categories, “response to heat” and “response to salt” were identified in the up-regulated gene list obtained from ice plants treated with 140 mM NaCl (Fig. 3B). In contrast, 6 and 10 GO categories were significantly enriched in the gene lists obtained from 250 mM and 500 mM NaCl-treated roots of ice plant, respectively (Fig. 3B). Although 31 GO categories were represented in the up-regulated genes identified in Arabidopsis treated with 140 mM NaCl, only five GO categories, “response to heat”, “response to cold”, “response to water deprivation”, “response to high light intensity” and “response to abscisic acid stimulus” overlapped with the GO categories that were identified for the up-regulated genes in *M. crystallinum* (Fig. 3B, bold text). Since only 44 genes were up-regulated in ice plant subjected to the 140 mM NaCl treatment, it was concluded that this level of salt does not have a large impact on the ice plant at the transcriptional level. The observation that the rate of root elongation in ice plants that were exposed to 140 mM NaCl was not significantly different than the rate in ice plants not subjected to salt stress (Fig. 1J), supports this contention. Regarding down-regulated genes, only the GO category “endomembrane system” was enriched in both the 500 mM NaCl-treated ice plant roots and the 140 mM NaCl-treated *Arabidopsis* roots (Fig. 3D, bold text). This may indicate that salt stress induces changes in the plant cell membrane system to protect cells from osmotic stress.

DREB2A is a key transcriptional regulator for salt response in *Arabidopsis* and other plant species and constitutive overexpression of DREB2A (DREB2A CA OX) resulted in a significant increase in salt tolerance [1]. To determine if *M. crystallinum* possesses a similar transcriptomic regulation to what is observed in DREB2A CA OX plants, the ice plant RNAseq datasets were compared to expression data obtained from the microarray analysis of DREB2A CA OX plants [1]. The up-regulation of four genes were found to be common amongst the DREB2A CA OX and 140 mM NaCl treated ice plant datasets. Additionally, those four genes were also up-regulated in the ice plant material that was treated with 250 mM and 500 mM NaCl (S1 Fig.). Lastly, 15 genes were up-regulated in both the 250 mM NaCl-treated ice plant roots and the DREB2A CA OX plants, and 21 genes were commonly up-regulated in both the 500 mM NaCl-treated ice plants and the DREB2A CA OX plants (S1 Fig. and S3 Table). Since only a few genes were commonly regulated in both the NaCl-treated ice plants and the DREB2 CA OX plants, it suggests that different regulatory mechanisms are involved in NaCl response in *M. crystallinum* and *Arabidopsis*, the latter of which is mediated by DREB2A.

### RT-qPCR confirmation of RNAseq results and differences in gene expression in the ice plant and *Arabidopsis*

**Fig 4:**
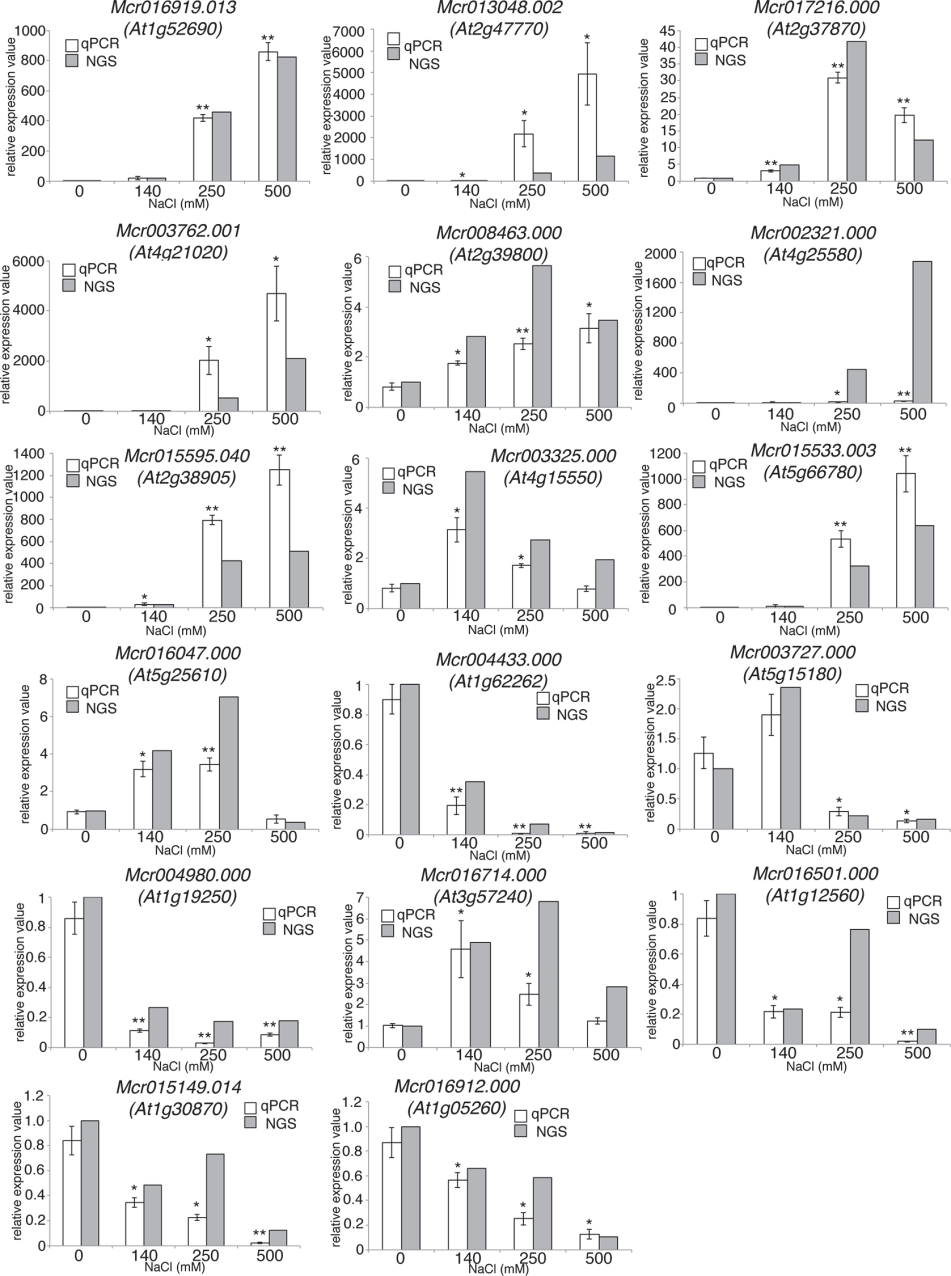
Comparison of gene expression data obtained by RNAseq and by RT-qPCR. Expression level of selected genes in whole roots of ice plant subjected to 0 mM, 140 mM, 250 mM, or 500 mM NaCl treatment for 24 h as measured by RT-qPCR (white boxes). Gene expression was normalized against the housekeeping gene, *PolyUBQ10*. (n = 3, ±S.D.; **and * indicate *p*<0.001 and *p*<0.05 by Student’s *t*-test, respectively, the comparison is between 0 mM NaCl and the remaining NaCl concentrations). Gray boxes (NGS) indicate the level of gene expression obtained from RNAseq data. The RNAseq data are shown as the level of expression relative to 0 mM NaCl treatment which was set at a value of 1.

RT-qPCR was used to confirm the RNAseq data, including the identification of genes and expression data. Twenty genes with significant changes in expression in at least one NaCl concentration were selected from the RNAseq data for confirmation by RT-qPCR (Fig. 4 and S2 Fig.). PCR primers were designed using the sequence data used to construct the contigs (S4 Table). Three of the twenty candidate genes exhibited no amplification. In contrast, sixteen out of the remaining seventeen genes tested exhibited the same level of expression profile in both the RNAseq and RT-qPCR results (Fig. 4). The expression levels were relative to that of *poly-UBQ10* and normalized to the value in 0 mM NaCl data, arbitrarily set as 1. The expression level of one of the twenty selected genes was different in the RNAseq and RT-qPCR data. The expression of Mcr002321.000, was much higher in the RNAseq data than the level indicated by RT-qPCR. Despite the expression differences obtained by the two methods for Mcr002321.000 transcripts were up-regulated by all salt treatments in both the RNAseq and RT-qPCR results. These results indicate that the expression dataset obtained for *M. crystallinum* using RNAseq was reliable for analyzing gene expression patterns.

Among the genes selected for confirmation by RT-qPCR, five were up-regulated by salt treatment in both the ice plant and *Arabidopsis*. Specifically, Mcr016919.013 (FC 22.8), which is an ortholog of a LEA family protein, and Mcr017216.000 (FC 3.2), which is an ortholog of a LTP family protein were up-regulated in DREB2A CA OX *Arabidopsis* plants overexpressing DREB2A [1]. Mcr016047.000, which is an ortholog of RD22, was up-regulated by salt treatments up to 250 mM NaCl in the ice plant, and also up-regulated in NaCl-treated *Arabidopsis*. Similar to FMO1 in *Arabidopsis*, Mcr004980.000, which is an ortholog of FMO1 [24], was strongly repressed by NaCl treatment in *M. crystallinum*.

Three genes (Mcr003727.000, Mcr015149.014, and Mcr016501.000) showed the opposite transcriptional response to NaCl treatment in ice plant than they did in *Arabidopsis* (S2 Fig.). Mcr016501.000, which is an ortholog of AtEXPA7, was repressed by NaCl treatment in ice plant but was up-regulated by 140 mM NaCl treatment in *Arabidopsis* roots (S2 Fig.). Mcr003727.000, which is an ortholog to peroxidase, was repressed by the 250 mM and 500 mM NaCl treatment and Mcr015149.014, which is an ortholog of a cationic peroxidase, were repressed in the ice plant but were up-regulated in *Arabidopsis* (S2 Fig.). Reactive oxygen species (ROS) metabolism is known to be involved in the NaCl response in *Arabidopsis* [25], and the GO category ‘peroxidase activity’ is enriched in *Arabidopsis* roots in a cell type-specific manner [7]. This also indicates that there are different mechanisms for responding to NaCl in *M. crystallinum* than in *Arabidopsis*.

## Discussion

Ice plant is a halophyte [12], and can survive in high salinity soils. The high levels of salt tolerance, present in some wild species of plants, present an excellent resource to study the adaptive mechanisms that form the basis of salt tolerance, and such plants may provide a valuable source of genes that can be used to improve salt tolerance in agronomic crops. In the present study, the transcriptional response of the ice plant, *M. crystallinum*, treated with different concentrations of NaCl was investigated using RNAseq. In the past ten years, high-throughput sequencing technologies have made whole genome sequencing of non-model organisms possible. As demonstrated in the present study, large numbers of short reads of transcripts of non-model organisms can be assembled into larger contigs composed of genes that can be identified, annotated, and quantified.

The genome size of *Arabidopsis* and rice is estimated to be 125 M bp and 389 M bp, respectively, with 28,517 and 37,869 encoded genes, respectively ([26], TAIR (http://www.arabidopsis.org/); [27], IRGSP1 (http://rapdb.dna.affrc.go.jp/)). The genome size of ice plant has been reported to be 250–300 M bp [13], [14], with an estimate of 30,000 to 35,000 genes. In the current study, 53,516 contigs were assembled and 10,818 of them were found to have orthologs in *Arabidopsis*. It has been suggested that 50–100 x coverage of the genome is required in order to assemble and analyze the genome of an organism by next generation sequencing [28]. Based on this estimate, approximately 25 G bp of sequence data would be needed to conduct a comprehensive analysis of the ice plant genome and that around 0.8 M bp of sequencing data will be required to identify a single gene. We obtained approximately 5 G bp of sequence data and identified about 11,000 genes with significant homology to *Arabidopsis* genes. This indicates that the amount of sequencing needed per gene is only about 0.4 M bp. Improved prediction of gene structure based on genomic sequences requires the ability of bioinformatic software to assemble contigs from short EST-like transcript sequences [29]. Since this requires a genome coverage of 50–100x, it is easy to see that RNAseq is an economic and efficient approach for investigating the transcriptome and genome of non-model organisms where a reference genome does not exist. The N50 of the ice plant contigs obtained in this study was 1,919 bp, which was larger than the N50 of 887 bp obtained for a semi-mangrove plant, *Millettia pinnata*, that was also obtained by RNAseq [11]. The N50 of *Arabidopsis* and rice was 1,809 and 1942 bp, respectively, which is very similar to what was obtained for ice plant, indicating that many of the contigs from ice plant would be expected to contain the sequence of nearly full-length transcripts.

Even with RNAseq technologies, there are difficulties in identifying genes that have a low level of expression. We identified 251 ice plant genes with homologues in *Arabidopsis* among the 644 genes that were significantly up-regulated by NaCl treatment in the *Arabidopsis* microarray datasets. The average expression level of these 251 genes, as determined by their signal intensity in the microarray, was approximately 322. The average gene expression level of the remaining 393 genes, for which homologous genes could not be identified in *M. crystallinum*, was 260. It is possible that all 393 of the genes that did not have homologues in the ice plant might represent genes that are unique to *Arabidopsis*. These results might be due to a failure in the ability to assemble the lowly expressed genes. A comparison of the expression data in the RNAseq datasets with the results obtained by RT-qPCR indicated that the results obtained by RNAseq were highly reliable and could be used to effectively characterize the genes that were affected by salt stress in the ice plant.

All results obtained in this study including the short reads, assembled sequences, and expression data have been deposited in a publically available database, along with some useful bioinformatic tools for analyzing the datasets. This database was established to foster collaboration between researchers and support present and future work on *M. crystallinum*.

Almost one-third of the genes identified in the ice plant have othologs in *Arabidopsis*. This number may not be sufficient enough to conduct a complete analysis of the gene regulatory network that is induced by salt in the ice plant. The GO analysis of the *Arabidopsis* dataset revealed a large number of GO categories that were up-regulated or down-regulated in response to the NaCl treatment. Almost all of the GO categories that were up-regulated in *Arabidopsis* were not identified in *M. crystallinum*. These results suggest that the system which allows ice plant to be salt tolerant is perhaps unique. Interestingly, “peroxidase activity” in ice plant was down-regulated by 250 and 500 mM NaCl but not in *Arabidopsis*. This GO category plays and important role in root growth, and the up-regulation of peroxidase activity in *Arabidopsis* has been reported to promote root growth [30]. The down-regulation of ‘peroxidase activity’-related gene expression in ice plant is consistent with the inhibition of root growth that was observed under the high salt concentrations. Moreover, this category was up-regulated by salt treatment in the *Arabidopsis* dataset, and the RT-qPCR result indicated that at least two peroxidase genes (At1g30870 and At1g05260) were regulated in an opposite manner in *Arabidopsis* vs. ice plant. Mcr016912.000, which is an ortholog of the *Arabidopsis* RCI3 peroxidase gene (At1g05260), decreased its expression level in ice plant in response to the NaCl treatment. Overexpressing *RCI3* in transgenic lines of *Arabidopsis* resulted in growth inhibition in response to salt stress [31]. A comparison of the transcriptome data of DREB2A CA OX with the transcriptome dataset of ice plant indicated that the number of genes significantly affected in both species was quite low. One gene in ice plant, a cationic peroxidase, which is an ortholog of At1g30870 in *Arabidopsis*, was strongly down-regulated but not in DREB2A CA OX (S2 Fig.). These data also indicate that ice plant likely uses a different mechanism than *Arabidopsis* in responding to salt stress. In addition to DREB2A, there are multiple transcription factors that are involved in salt tolerance (see review in [32], [33], [34]). For the next step in the study of salt tolerance mechanisms, we will be able to use our datasets for comparing other signals that are regulated by other transcription factors besides DREB2A orthologs. It is plausible to suggest that perhaps the salt tolerance genes identified in the *Arabidopsis* studies are already expressed at a high level in ice plant even when the plant is not exposed to salinity. For this reason, ice plant exhibited tolerance to the 140 mM NaCl treatment and roots were able to continue to grow.

On the other hand, several genes analyzed in the current study exhibited the same expression response to NaCl in both *Arabidopsis* and the ice plant. However, only five GO categories, “response to heat”, “response to cold”, “response to water deprivation”, “response to high light intensity” and “response to abscisic acid stimulus” were up-regulated in both *Arabidopsis* and in ice plant. Salt treatment has been reported to increase endogenous ABA levels in the roots of ice plant [35]. This finding, along with our transcriptome data, indicates that ABA plays an important role as a signal molecule in the response of both the ice plant and *Arabidopsis* to salt stress. A QTL analysis of salt tolerance in *Arabidopsis* was conducted by Katori et al. [9] to identify loci that are associated with salt tolerance. They examined 350 accessions of *A. thaliana*. One accession (Bu-5) was used for the transcriptome analysis and it was found that Δ-1-pyrroline-5-carboxylate synthetase 1 (P5CS1; At2g39800) was up-regulated in this accession and other accessions exhibiting salt tolerance. P5CS is an enzyme which regulates a rate-limiting step in proline biosynthesis [36]. A previous report also demonstrated that proline accumulates in ice plant roots in response to salt treatment [37]. In addition, we also found that a P5CS1 ortholog was up-regulated in ice plant (Fig. 4, S2 Fig.).

Additionally, a genome wide association study (GWAS) of *Arabidopsis* accessions identified one locus, which possessed a gene encoding a sodium transporter protein, HKT1 [10]. Allelic variation in this gene was reported to be a major factor responsible for the natural variation in the ability to accumulate Na in leaves and salt tolerance in general [10]. Agarie et al. [38] reported that the mechanism responsible for salt tolerance in the ice plant is its ability to transport salt from roots to the shoots, where it accumulates in bladder cells on the surface of leaves. In our study, the AtHKT1;1 ortholog in ice plant was significantly down-regulated in response to NaCl treatment (S1 Table). The similar result on AtHKT1;1 expression observed in *Arabidopsis* and the ice plant in response to salt treatment, suggests that the ability to transport and isolate excess amounts of Na also plays an important role in salt tolerance in ice plant.

In conclusion, a comprehensive transcriptome analysis of the response to salt of the ice plant, *M. crystallinum*, was conducted and the resulting dataset was compared with *Arabidopsis* gene expression data obtained from previous studies using microarray. Using these data, we provided an overview of gene expression in the two species in response to salt stress and how expression was either similar or different. *M. crystallinum* is not a commonly used model plant species and a sequenced reference genome is not available. Using our transcriptomic datasets, however, we were able to observe new patterns of gene expression associated with salt tolerance in the ice plant and identify the sequence of the genes associated with salt tolerance in ice plant. Transgenic approaches can now be used to conduct functional studies of these ice-plant-specific genes in model plants and economically important crop species. Furthermore, metabolomic and proteomic data can be combined with our transcriptomic data to develop a comprehensive understanding of salt tolerance in *M. crystallinum*.

## Supporting Information

S1 Table Gene expression data based on the RNAseq analysis of the transcriptome of ice plant roots treated with various concentrations of NaCl. (XLSX)

S2 Table Gene expression in roots of NaCl-treated *Arabidopsis* plants. Data were obtained from the *Arabidopsis* microarray database (http://www.ncbi.nlm.nih.gov/gds/).
(XLSX)

S3 Table Levels of gene expression in roots of DREB2A CA OX plants and ice plants in response to salt stress. (XLSX)

S4 Table The list of the primers used to conduct an RT-qPCR analysis of gene expression in ice plant root exposed to salt stress. (XLSX)

S1 Fig Venn diagram illustrating the overlap of salt-responsive gene expression in DREB2A CA OX and ice plants. Data on gene expression in DREB2A CA OX were obtained from the published study by Sakuma et al. (2006) and data on the ice plant was obtained from the RNAseq data in the present study. The Venn diagram only displays those genes that were up-regulated.
(TIF)

S2 Fig Comparison of gene expression patterns in ice plants, *Arabidopsis* (ecotype Col-0), DREB2A CA OX, and *Arabidopsis* (ecotype Bu-5). The heat map indicates fold change (log2 scale) compared to no-salt (control) conditions. Bu-5 data were obtained from the published study by Katori et al. (2010). * indicates FDR to be consistent q<0.05 that were calculated by R package, DESeq.
(TIF)
