# Supplementary figures and images for: RNA-Seq Analysis of the Response of the Halophyte, Mesembryanthemum crystallinum (Ice Plant) to High Salinity
Source: PLoS One. 2015 Feb 23;10(2):e0118339. doi: 10.1371/journal.pone.0118339 (PMC4338230; doi:10.1371/journal.pone.0118339)

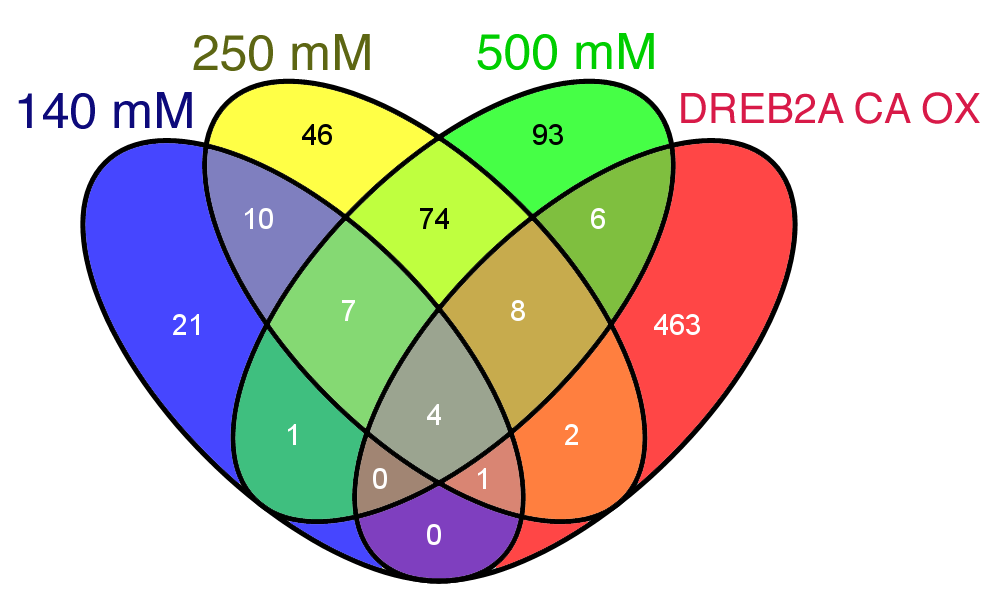

Supplement: S1 Fig — Data on gene expression in DREB2A CA OX were obtained from the published study by Sakuma et al. (2006) and data on the ice plant was obtained from the RNAseq data in the present study. The Venn diagram only displays those genes that were up-regulated. (TIF) [file pone.0118339.s005.tif]

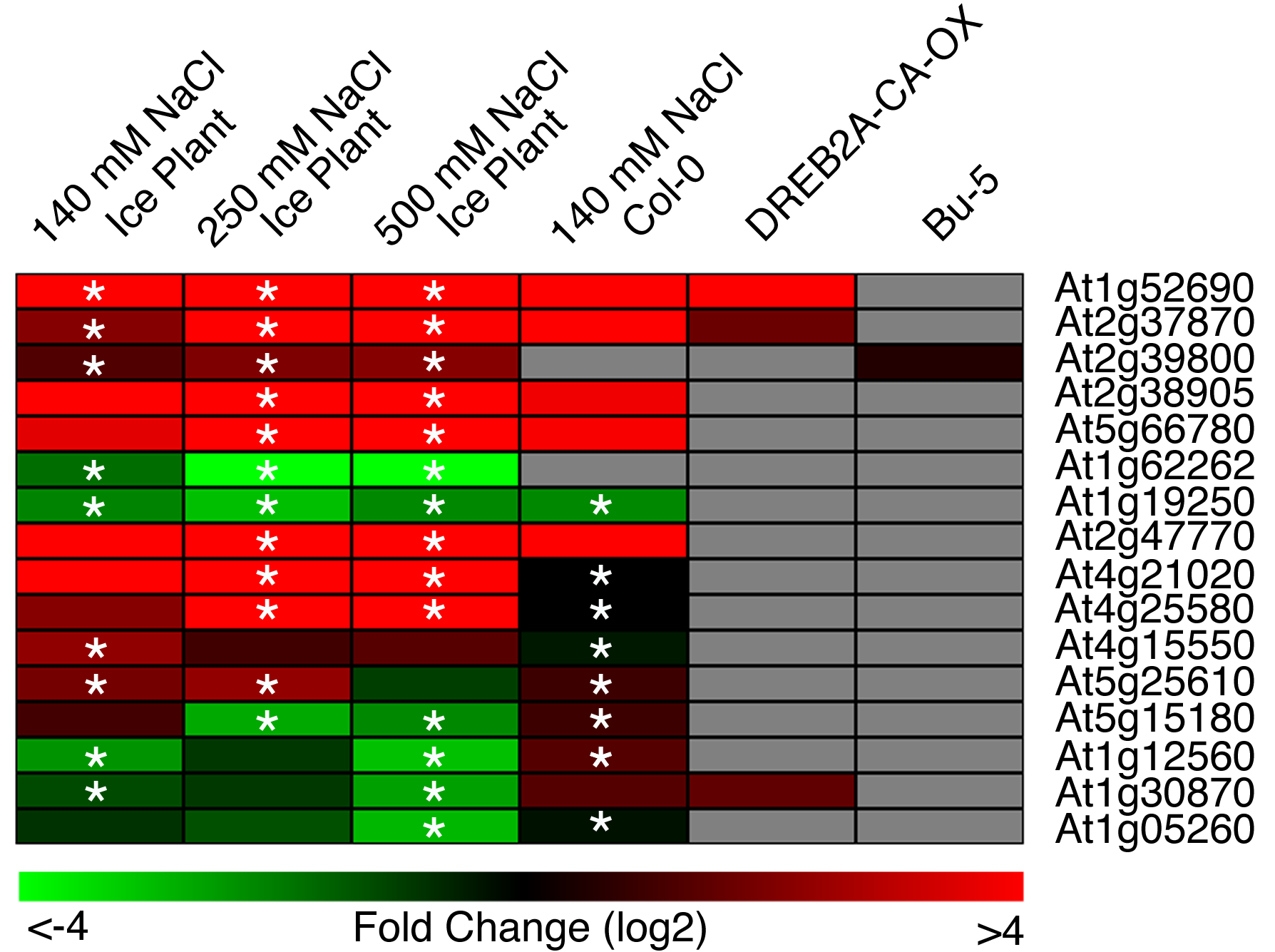

Supplement: S2 Fig — The heat map indicates fold change (log2 scale) compared to no-salt (control) conditions. Bu-5 data were obtained from the published study by Katori et al. (2010). * indicates FDR to be consistent q<0.05 that were calculated by R package, DESeq. (TIF) [file pone.0118339.s006.tif]
